# Multispecies Coalescent Analysis of the Early Diversification of Neotropical Primates: Phylogenetic Inference under Strong Gene Trees/Species Tree Conflict

**DOI:** 10.1093/gbe/evu244

**Published:** 2014-11-05

**Authors:** Carlos G. Schrago, Albert N. Menezes, Carolina Furtado, Cibele R. Bonvicino, Hector N. Seuanez

**Affiliations:** ^1^Department of Genetics, Federal University of Rio de Janeiro, Brazil; ^2^Division of Genetics, National Cancer Institute, Rio de Janeiro, Brazil; ^3^Laboratory of Biology and Parasitology of Wild Mammal Reservoirs, Instituto Oswaldo Cruz, Fiocruz, Brazil

**Keywords:** species tree, primate evolution, coalescence, South America, Miocene

## Abstract

Neotropical primates (NP) are presently distributed in the New World from Mexico to northern Argentina, comprising three large families, Cebidae, Atelidae, and Pitheciidae, consequently to their diversification following their separation from Old World anthropoids near the Eocene/Oligocene boundary, some 40 Ma. The evolution of NP has been intensively investigated in the last decade by studies focusing on their phylogeny and timescale. However, despite major efforts, the phylogenetic relationship between these three major clades and the age of their last common ancestor are still controversial because these inferences were based on limited numbers of loci and dating analyses that did not consider the evolutionary variation associated with the distribution of gene trees within the proposed phylogenies. We show, by multispecies coalescent analyses of selected genome segments, spanning along 92,496,904 bp that the early diversification of extant NP was marked by a 2-fold increase of their effective population size and that Atelids and Cebids are more closely related respective to Pitheciids. The molecular phylogeny of NP has been difficult to solve because of population-level phenomena at the early evolution of the lineage. The association of evolutionary variation with the distribution of gene trees within proposed phylogenies is crucial for distinguishing the mean genetic divergence between species (the mean coalescent time between loci) from speciation time. This approach, based on extensive genomic data provided by new generation DNA sequencing, provides more accurate reconstructions of phylogenies and timescales for all organisms.

## Introduction

The anthropoid primate infraorder Platyrrhini presents remarkable morphologic, ecologic, and behavioral variation (Fleagle 2013). Platyrrhines are distributed in the neotropical forests of Mexico, Central, and South America, comprising a sister clade of Old World anthropoids (catarrhines). Molecular dating estimates indicated that neotropical primates (NP) diverged from catarrhines near the Eocene/Oligocene boundary, some 40 Ma (Schrago 2007; Perelman et al. 2011; Schrago et al. 2012) when South America was an island continent separated from Africa. Since no anthropoid fossil was ever found in North America or Antarctica, the most likely NP ancestors must have originated in the African continent where an extensive fossil record of primitive anthropoids resembling NP has been found (Fleagle 2013). This hypothesis necessarily assumes that ancestral NP reached the South American island continent by transoceanic dispersal, via rafting, or island hopping (Houle 1999; de Queiroz 2005).

Apart from their geographic origin and dispersal, the evolutionary divergence of NP has been controversial. Several authors have suggested that the age of the most recent NP ancestor can be traced back to approximately 20 Ma (Schrago 2007; Kay et al. 2008; Perelman et al. 2011; Schrago et al. 2013). This implies that, if platyrrhine and catarrhine anthropoids split approximately 40 Ma (Perelman et al. 2011), several early NP lineages that emerged during the initial 20 Myr must have become evolutionary dead-ends without involvement in the radiation of the major, extant NP lineages, comprising the families Cebidae, Atelidae, and Pitheciidae (Schneider 2000). Moreover, the phylogenetic relationships between the three NP families are not fully clarified because recently proposed associations between Cebidae and Atelidade have not been strongly supported (Hodgson et al. 2009; Perelman et al. 2011).

These unresolved issues associated with the tempo and mode of NP evolution require a well-resolved phylogeny and timescale in order to be properly addressed. With this respect, several timescales of NP diversification have been proposed in the last decade (Opazo et al. 2006; Schrago 2007; Hodgson et al. 2009; Perelman et al. 2011) but these timescales, however, were based on limited numbers of loci and dating analyses that did not consider the evolutionary variation associated with the distribution of gene trees within the proposed phylogenies. This association is crucial for distinguishing the mean genetic divergence between species (the mean coalescent time between loci) from speciation time (Edwards and Beerli 2000). A similar situation exists for the well-known age of genetic isolation between humans and chimpanzees, which is considerably more recent than the mean age of the genetic divergence between their loci (Hobolth et al. 2007; Prado-Martinez et al. 2013).

Analyses of data sets consisting of thousands of loci are capable of estimating parameters associated with the topological and temporal variation between gene trees for estimating the species tree (Edwards 2009). Several studies have shown that phylogenetic inference may be biased if reconstruction methods fail to account for the natural variation of the phylome, or the collection of gene trees inferred along genomes (Edwards et al. 2007; Liu et al. 2009). Song et al. (2012), for example, reported that analysis of concatenated supermatrices with maximum likelihood (ML) and Bayesian methods produced consistently different topologies of mammalian orders when compared with estimates based on coalescent-based methods.

In this study, we analyzed a large collection of molecular markers of representative species of the three major NP lineages following next-generation sequencing of two primates: *Brachyteles arachnoides* (muriqui), the largest-sized living primate endemic to the Americas, with a diploid chromosome number of 62, and *Callicebus lugens*, a titi monkey endemic to the Western Amazon with the lowest diploid chromosome number (2n = 16) found in the primate order (Bonvicino et al. 2003). These data, together with the publically available genome of the marmoset (*Callithrix jacchus*) accounted for representative genomes of the major evolutionary NP lineages. By employing a multispecies coalescent analysis, we contribute to clarifying why molecular analyses for resolving the evolutionary affinities between the major NP lineages have been so controversial along the last 20 years. We herein show that the early diversification process of extant NP occurred very rapidly and took place in an ancestral population with a very large effective size. We also show that the gap between the genetic divergence of New and Old World anthropoids, and the age of the first platyrrhine record in South America might have resulted from an incorrect equivalence of mean coalescent time and speciation time, a finding that must be accounted for historical biogeography.

## Materials and Methods

### Samples

#### Collection of Samples and Ethical Considerations

Peripheral blood samples were obtained from a captive, male *B. arachnoides* (CPRJ2506) kept in the Centro de Primatologia do Rio de Janeiro (CPRJ-INEA). Samples used in this study were part of the blood samples regularly collected for checkups and control of captive animals. Liver tissue from one wild caught female *C. lugens* (Field number CRB 2677). Sample collections were carried out following the national guidelines and provisions of IBAMA (Instituto Brasileiro do Meio Ambiente e dos Recursos Naturais Renováveis, Brazil; permanent license number 11375-1). The granting of this licence by IBAMA followed approval by its Ethics Committee. The Centro de Primatologia do Rio de Janeiro is located 100 km northeast of the city of Rio de Janeiro, in a protected forest area of the Serra dos Órgãos mountain range. The Centro is not open to visitors. In this facility, animals are housed in groups in outdoor enclosures consisting of wire mesh, being exposed to the Atlantic Forest conditions such as sounds, temperature, and rainfall. The *Brachyteles* enclosures measure 16.0 m × 6.0 m × 6.0 m high with an additional area of 2 m × 6 m × 3 m high for feeding. Food and fresh water were provided twice a day. The diet consisted of bread, bananas, eggs, raisins, meat, and various commercially prepared protein supplements.

#### Genome Sequencing

Genomic DNA of *B. arachnoides* was extracted with QIAamp DNA Mini and Blood Mini kit (Quiagen and fragmented with an Invitrogen nebulizer under 40 psi for 40 s in TE buffer and 25% glycerol generating 300–400 bp fragments observed by electrophoresis. DNA from *C. lugens* liver tissue was extracted with phenol chloroform (Sambrook et al. 1989). Sample quality was checked by both electrophoresis in agarose 1% gels and NanoDrop 1.000 Spectrophotometer (Thermo Scientific) and quantified using a Qubit 2.0 Fluorometer (Life technologies).

Library preparation for *B. arachnoides* DNA followed Illumina Sequencing Workflow protocols with TrueSeq DNA Sample Prep (Illumina) for the HiSeq2000 platform. DNA containing 400–500 bp fragments, checked with Agilent Bioanalyzer 2100 DNA using a high sensitivity DNA chip, was subsequently tested by quantitative polymerase chain reaction (qPCR) using a Library Quantification kit for library validation (KAPA–KK4824). DNA Cluster generation was prepared for two lanes of a PE Flowcell v.3 following the manufacturer’s protocol. A 75 × 75 run was carried out in an Illumina HiSeq2000 platform. Cycles registered 84.4% base calls with Q30 quality score.

Library preparation of *C. lugens* DNA followed the Illumina Sequencing Workflow protocols (Nextera Kit or Truseq DNA Sample Prep) for the HiSeq2000 platform. DNA containing 800–900 bp fragments (with Nextera kit) and 400–500 bp (with Truseq kit), checked with Agilent Bioanalyzer 2100 DNA with a high sensitivity DNA chip, was subsequently tested by qPCR using a Library Quantification kit for library validation (KAPA–KK4824). DNA cluster generation was prepared for three lanes of a PE Flowcell v.3 for *C. lugens* following the manufacturer’s protocol. A 101 × 101 pairend run was carried out in an Illumina HiSeq2000 platform (with a minimum of 83.6% registered base calls with Q30 quality score).

#### Genome Assembly and Search for New Molecular Markers

**Fig. 1.—:**
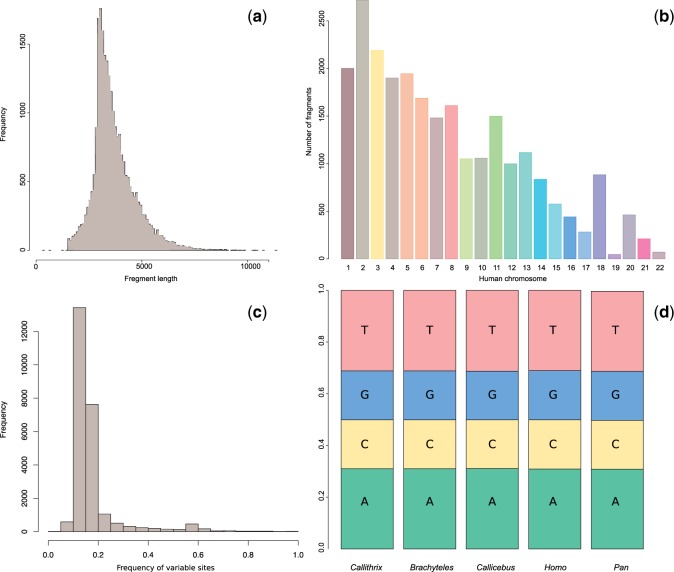
(*a*) Size distribution of orthologous alignments; (*b*) distribution of orthologous alignments with human chromosomes; (*c*) distribution of the frequency of variable sites per alignment used; (*d*) average frequency of nucleotides on each species analyzed.

Genome assembly was conducted de novo with ABySS assembler using paired end libraries (Simpson et al. 2009). For each genome, assembly performance was optimized by scanning the best *k*-mer value from 25 to 55. Genome scaffolds were subsequently aligned against database of syntenic alignments from *Homo sapiens*, *Pan troglodytes*, and *Callit. jacchus* genomes available in the Ensembl’s Compara repository (ftp://ftp.ensembl.org/pub/release-67/emf/ensembl-compara; last accessed October 9, 2014) using the LAST software (Kielbasa et al. 2011). These newly generated syntenic alignments were subsequently scanned to obtain regions with small amounts of indels and missing data (fig. 1), and with full taxonomic coverage, resulting in 25,955 genome segments of approximately 3,500 bp. The final concatenated supermatrix of selected genome segments consisted of 92,496,904 bp. The length of genome segments was determined by scaffold length, and orthologous regions were identified by LAST. The average frequency of variable sites was 17.5% (standard deviation = 10.3%) and the percentage of nucleotides was very homogeneous between species (fig. 1).

Because multispecies coalescent methods rely on constant evolutionary rates along branches, we have also analyzed a data set comprising the genomic segments that fitted with the molecular clock. Each of the 25,955 genomic segments was subjected to a molecular clock test using a likelihood ratio test (LRT) with a significance level of 5% (Felsenstein 1988). As estimates obtained from the segments fitting the molecular clock were identical to estimates from the full data set, this latter was used for further analysis.

#### Rationale for Comparative Analyses

Molecular markers of *B. arachnoides,* a representative species of the Atelidae, *C. lugens*, a representative species of the Pitheciidae, *Callit. jacchus*, a representative species of the Cebidae (The Marmoset Genome Sequencing and Analysis Consortium 2014), *H. sapiens* (Lander et al. 2001; Venter et al. 2001), and *P. troglodytes* (Chimpanzee Sequencing and Analysis Consortium 2005) were used in these analyses.

After compiling alignments of orthologous sequences, each molecular marker was analyzed independently and in a concatenated supermatrix. This supermatrix was also used for inferring mean genetic divergence times between lineages, that is, gene divergences. Coalescent-based analyses were subsequently conducted for deriving the empirical distribution of gene tree topologies and estimating ancestral, effective population sizes, and speciation times, that is, population divergence estimates.

#### Gene trees, Divergence Times, and the Species Tree

A gene tree for each genomic segment was inferred by ML with PhyML 3 (Guindon et al. 2009) using the substitution model chosen by the LRT with the modeltest batch file of HyPhy (Pond et al. 2005), with significance level set at 5%. The tree topology of the concatenated supermatrix was also inferred with PhyML following the same procedure. Divergence times, based on gene divergence between lineages rather than speciation times, were inferred using the relaxed clock approach via the approximate likelihood method implemented with PAML’s mcmctree program (Yang 2007). In mcmctree, the posterior distribution of node ages and evolutionary rates were estimated using the Markov chain Monte Carlo (MCMC) algorithm, sampling the Markov chain every 100th cycle until obtaining 30,000 samples. A burn-in period of 10,000 generations was adopted and analyses were run twice for confirming convergence. The prior rate distribution was modeled by the uncorrelated lognormal distribution. Information for calibrating the relaxed clock was entered as two prior distributions for node ages. First, the *Homo–Pan* separation was calibrated by a normal prior with a mean of 8.25 Ma and standard deviation of 0.9 Myr. This prior was based on Benton and Donoghue (Benton and Donoghue 2007); the mean being the average between the maximum and minimum ages of lineage divergence, and the standard deviation adjusted to containing minimum and maximum values within the 99% highest probability density (HPD) interval. The root of the tree, at the platyrrhine/catarrhine divergence, was modeled by a normal prior with a mean of 40 Ma and standard deviation of 7 Myr. This was a flexible prior allowing the age of the basal anthropoid node to vary from 27 Ma, the age of the earliest platyrrhine fossil *Branisella boliviana* (Takai and Anaya 1996), to 60 Ma, the age of early primates (Hartwig 2002).

Estimation of the species tree was conducted with MP-EST and STAR (Liu and Yu 2010) algorithms implemented in the STRAW webserver and with the concordance factor analysis implemented in BUCKy (Ané et al. 2007). MP-EST and BUCKy methods produced species trees with branch lengths measured in coalescent units (number of 2*N*_e_ generations). As a means of investigating the relative support of alternative phylogenetic groupings of NP families, we have also derived the distribution of statistical supports, namely, the Bayesian posterior probabilities and the approximate LRT (aLRT) (Anisimova and Gascuel 2006) for each alternative clade (see below). For species tree- and other coalescent-based analyses, we eliminated the chimpanzee sequence and used *Homo* as outgroup. This allowed us to study the standard, four-species topological space that permits a better exploratory analysis while saving computational time.

The distribution of Bayesian posterior probabilities for each clade was obtained by parsing MrBayes’trprob output files produced independently for each gene. For each gene, whenever a topology containing a given clade was not sampled during MCMC run, a zero posterior probability was attributed. The distribution of aLRTs was obtained by simply collecting the aLRT clade support in the ML tree inferred for each gene. For instance, we computed the Bayesian posterior probability of the (*Brachyteles*, *Callithrix*) clade for all genes herein analyzed, even for genes producing trees in which this clade was not sampled in MCMC, that is, posterior clade probability = 0. On the other hand, aLRTs for this clade were collected only from ML trees that contained this group.

We have also estimated the number of coalescent units between the age of the last common ancestor (LCA) of living NP and the speciation time of atelids and cebids. As the number of genome segments studied was very large, the empirical distribution of gene tree topologies was assumed to approach the theoretical distribution of gene trees within a given species tree. The probability of topological matching between the species tree and the gene tree is given by

$$P=1-\frac{2}{3}e^{-\frac{\Delta t}{2{\text{N}}_{\text{e}}}}$$

(Pamilo and Nei 1988). In this formula, Δt is the time length of the internal branch measured in generations. A substitution of *P* by the frequency of inferred gene trees matching the species tree topology allows for estimating $\Delta_{t}$ in 2*N*_e_ generation units. It is worth mentioning that this formula calculates the topological mismatch between gene trees and the species tree for a rooted, three-species association. Thus, individual gene trees estimated by ML or BI were rooted using *Homo.*

#### Inference of Ancestral Effective Population Size

Inference of ancestral population size was performed with the BPP program, implementing the Bayesian method of Rannala and Yang (2003). This method also used MCMC simulation for deriving the posterior distribution of population sizes and speciation times. In BPP, both population sizes (θ) and speciation times (τ) are scaled by the yearly mutation rate (μ), thus ${\theta =\text{4}N}_{e}\mu \text{g}$ and τ=tμ, where *g* is the generation time and *t* is the speciation time in years. A burn-in period of 20,000 generations was used and 50,000 samples were later collected from Markov chains every tenth step. The prior distributions of effective population sizes were modeled by the gamma prior *G*(α = 2, β = 200). The prior distribution of root age, referring to the time (as number of substitutions per site) to the complete isolation of platyrrhines from catarrhines, was modeled by a gamma prior. This gamma prior was approximated with the MASS package of R software, using the posterior distribution of the product of node ages (in years) and mean evolutionary rates (substitutions/site/year) obtained in the mcmctree program as described above. Transformation of scaled effective population sizes into absolute number of individuals requires estimates of ancestral generation times and the yearly evolutionary rate. Ancestral generation times were inferred using the approach described in Schrago (2014). The yearly evolutionary rate was set at the value of the mean evolutionary rate, which was estimated as previously described.

#### Calculation of Gene Tree Probabilities

Estimates of ancestral effective population sizes, speciation times, and generation times were used for calculating the probabilities of gene trees given the species tree (Degnan and Salter 2005). The theoretical distribution of gene trees for a given species tree was compared with the empirical collection of gene trees inferred from several loci across genomes. If estimates of ancestral population sizes were correct, the theoretical distribution of gene trees would be expected to closely match the empirically estimated gene trees. The theoretical distribution of gene trees was obtained with COAL 2.1, requiring the input of a species tree with internal branches measured in coalescent units resulting from dividing branch (in generations) by twice the effective population size (Degnan and Rosenberg 2009). Ancestral population sizes and speciation times were previously estimated with BPP.

## Results

**Fig. 2.—:**
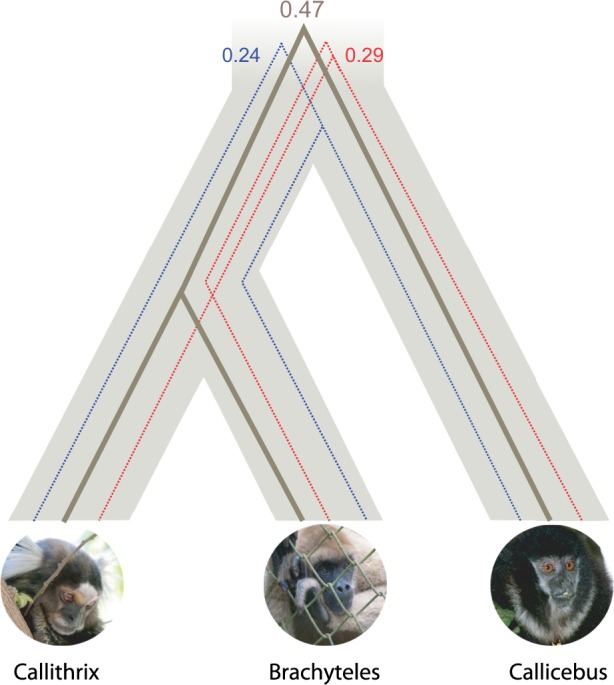
Frequency of gene tree topologies within the inferred species tree.

**Fig. 3.—:**
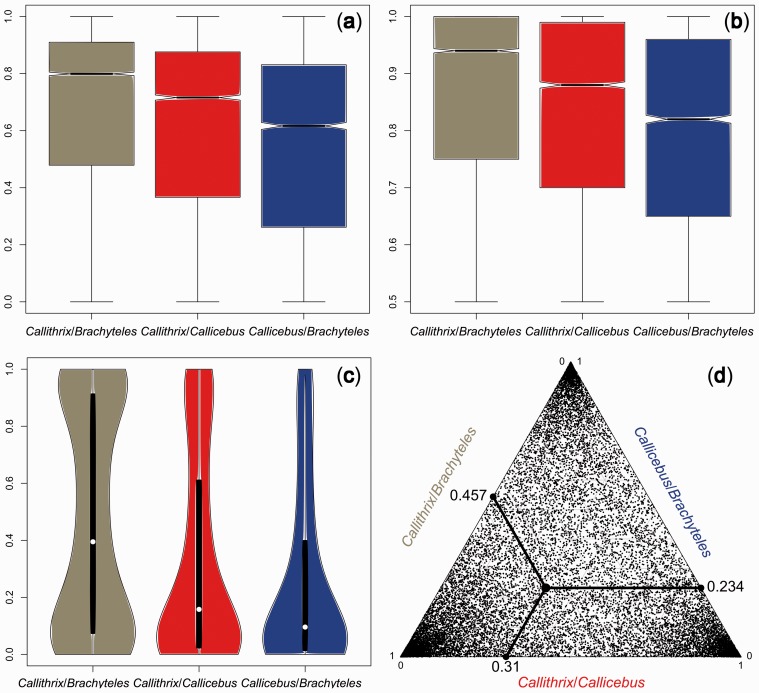
Distribution of statistical supports of topological NP arrangements. (*a*) Distribution of aLRT supports along gene trees; (*b*) distribution of posterior clade probabilities along consensus Bayesian gene trees; (*c*) violin plot showing distribution of the Bayesian posterior probabilities for the full topologies. In (*d*), each point of the triangle plot depicts the posterior probability of full topologies for each gene tree. Average values are highlighted.

Coalescent-based phylogenetic analyses inferred the *Brachyteles*/*Callithrix* topology as the NP species tree (fig. 2). The majority of ML and Bayesian inferred gene trees (∼47%) supported a topology with *Brachyteles* as the sister lineage of *Callithrix* whereas the second most common topology associated *Callithrix* with *Callicebus* (29%), followed by the association of *Brachyteles* and *Callicebus* (24%). The estimated length of the branch separating the *Callicebus* offshoot from *Brachyteles/Callithrix* varied from 0.255 to 0.447 coalescent units (table 1). The traditional ML method and Bayesian inference also positioned *Brachyteles* as a sister lineage of *Callithrix* when a concatenated supermatrix of all genes was used. Bayesian concordance analysis inferred a concordance factor of 0.541 for the *Brachyteles*/*Callithrix* topology, whereas both alternative topologies showed concordance factors higher than 5%: 0.255 for the *Callithrix*/*Callicebus* tree and 0.204 for *Brachyteles*/*Callicebus*. The distributions of statistical supports for each topology along gene trees also indicated a high gene tree/species tree conflict. For example, the average aLRT support for the *Brachyteles*/*Callithrix* clade was approximately 80% (fig. 3), with an average Bayesian posterior probability for this clade of approximately 95%. The distribution of Bayesian posterior probabilities of full topologies also showed a large amount of mismatching between gene trees and the species tree. The average posterior probability of topologies matching the species tree accounted for 46% (fig. 3*d*).

**Table 1:** Estimates of the Time Interval between the Age of the LCA of Living NP and the Age of the Atelidae/Cabidae Node

Method	$\Delta_{t}$ (2*N_e_* Generations)
MP-EST	0.256
BUCKy	0.374
Analytical (Bayes, consensus)	0.263
Analytical (Bayes, MPP)^a^	0.447
Analytical (ML)	0.255

^a^Maximum posterior probability topology >95%.

**Fig. 4.—:**
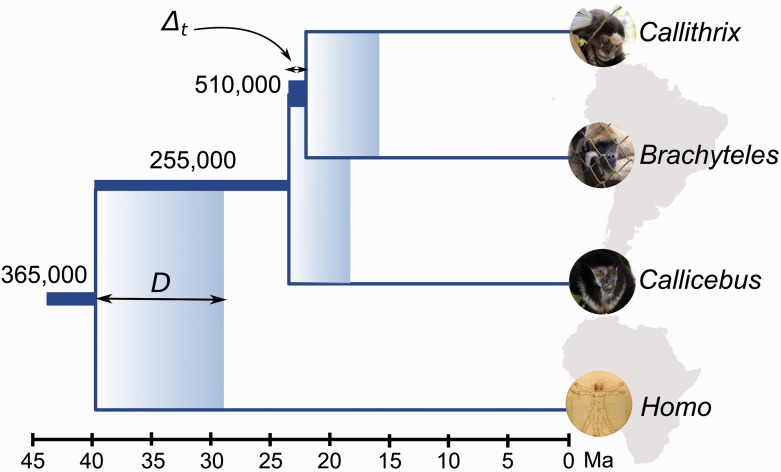
Timescale of the early NP diversification. $\Delta_{t}$ refers to the time interval between the LCA of living NP and the diversification time between atelids and cebids. *D* indicates the difference between genetic divergence time and speciation time. The width of internal branches is proportional to the effective size of ancestral populations, which are shown above branches.

The average genetic divergence between New World and Old World anthropoids was inferred as 38.4 Ma, with 95% HPD interval ranging from 26.9 to 54.7 Ma. The age of the LCA of extant NP was estimated as 22.9 Ma (15.8–33.0) (fig. 4). The split between the Cebidae and Atelidae occurred roughly 1 Myr after the basal diversification of living platyrrhines, at 21.5 Ma (15.0–31.3). The average rate of molecular evolution was inferred as 1.4 × 10^−^^9^ substitutions/site/year (s/s/y). Assuming this rate, the age of complete genetic isolation between cebids and atelids was inferred at 15.2 Ma and the age of the LCA of living NP at 17.2 Ma. Finally, the complete genetic isolation between platyrrhines and catarrhines occurred at 27.5 Ma (table 2).

**Table 2:** BPP Estimates of Scaled Effective Population Sizes, Absolute Effective Population Sizes, Divergence Times, and Speciation Times, μ = 1.4 × 10^−9^ s/s/y and *g* = 10.3 Years

Node	θ = 4*N*_e_μg	*N*_e_	*T*	τ = *t* μ
Atelidae/Cabidae	0.02774	267,857–750,000	21.5	15.2
Crown Platyrrhini	0.01258	150,000–357,143	22.9	17.2
Platyrrhini/Catarrhini	0.01922	250,000–482,140	38.4	27.5

The formula that relates the probability of gene tree/species tree topological concordance with effective population size indicated that the common ancestor of cebids and atelids accounted for 250,000 individuals. This was estimated assuming an average generation time of 10.3 years for the ancestral NP population. The scaled effective population size (θ) of the cebid–atelid ancestor inferred by BPP was estimated as 0.02774, whereas the scaled effective size of the ancestor of all living platyrrhines was inferred as 0.01258. Finally, at the root node (the split between platyrrhine and catarrhine anthropoids) the scaled affective population size was estimated as 0.01922. Assuming an evolutionary rate of 1.4 × 10^−^^9^ s/s/y and generation time of 10.3 years, the 95% HPD interval of the *N*_e_ estimate of the common ancestor of cebids and atelids varied from 267,857 to 750,000. The *N*_e_ estimate shifted to 150,000–357,143 at the crown NP node and subsequently increased to 250,000–482,140 individuals at the basal anthropoid node. These estimates showed that early diversification of extant NP was marked by a 2-fold increase of their effective population size.

## Discussion

Our results showed that the evolutionary relationships of NP are difficult to reconstruct because of the high proportion of gene trees/species tree incongruence between the three main extant platyrrhine families. Along the history of New World primates, effective population size increased since the early diversification of extant lineages. This largely explains the low probability of matching gene trees with species trees. Moreover, the 2-fold increase of the effective population size within a period of approximately 1 Myr indicated that radiation of extant lineages was clearly a rapid and explosive event.

The timing of the major nodes implies that living NP are descendants of an early Miocene ancestor and that family level cladogenetic events also occurred in this period. This scenario was recovered independently of analytical methods. The inferred times of the complete genetic isolation of sister lineages (τ) also pointed to a rapid diversification scenario of the three major NP families and all estimates were in agreement with the fossil record. Ages inferred by the Bayesian clock, representing the mean genetic divergence (*T*) and from coalescent-based analysis (τ), suggested that the LCA of extant platyrrhines was older than 15.7 Ma, which is the age of *Proteropithecia*, the oldest, undisputedly assigned fossil to any living NP family (Pitheciidae) (Kay et al. 2008), representing the first offshoot of living NP. Moreover, the age of full genetic isolation (τ) is expected to be closer to the timescale of the fossil record. This was the case of the inferred ages of the cebid–atelid divergence supported by the finding of the stem cebid *Mohanamico hershkovitzi* and *Stirtonia*, the oldest undisputed atelid, 12.2 and 13.6 Ma, respectively (Hartwig 2002). The recent finding of a putative crown platyrrhine in the Peruvian Amazonia at 16–17 Ma (Antoine et al. 2012) is also in agreement with our estimates. It is worth mentioning that, although the age of the crown-living platyrrhines inferred by the Bayesian clock was enforced to be older than 15.7 Ma, this constraint was not implemented in the coalescent-based analysis. The estimation of τ values, in accordance with paleontological data, further corroborated our proposal.

The early diversification of living NP largely overlaps the period in which the South American continent elapsed through periods of warmer climate during the Miocene (23–17 Ma, the Mid-Miocene Climate Optimum), after the reduction of sea level, associated with the opening of a southeastern gateway and retraction of the Paranense Sea in the South American continent. It was also a period of intense geological modifications in the northern part of South America resulting from the uplift of the central and northern Andes mountain range, disruption of the sub-Andean river system and onset of the Pebas wetland system (Hoorn et al. 2010). Six out of 16 NP genera are endemic to the Amazon, which reinforces the claim that this region is pivotal to comprehend the evolution of extant NP. We therefore postulate that the rapid rise of modern NP families was closely related to this phase (Delsuc et al. 2004).

The age of the average genetic divergence between New World and Old World anthropoids was dated at the Late Eocene (∼38 Ma), corroborating recent reports (Perelman et al. 2011; Springer et al. 2012; Schrago et al. 2013). Our estimates of the age of the complete genetic isolation of both lineages was, nevertheless, significantly younger, approximately 27.5 Ma. This estimate was validated by the fossil record because the age of the oldest NP fossil, *Bran. boliviana*, is approximately 26 Ma (Takai et al. 2000). In this respect, the difference between the mean genetic divergence between catarrhines and platyrrhines (∼38 Ma) and speciation time (27.5 Ma) may clarify the time gap between the age of the mean genetic divergence and the age of the earliest primate fossil in the Neotropics. The hypothesis of a “ghost” lineage in this period is likely an artifact consequently to considering the age of the mean genetic divergence equivalent to the age of genetic isolation. As paleontological efforts in South America increases, it might be argued that the fossil record of earliest NP may be extended further back to approximately 40 Ma because of the recent finding of a hystricognath rodent in Late Eocene deposits (Antoine et al. 2012). This conclusion relies on the hypothesis that ancestors of NP and hystricognaths invaded South America synchronously. However, comparative molecular dating analyses of the diversification of platyrrhines and hystricognath rodents demonstrated that the separation of New World and Old World Hystricognathi took place before the divergence between catarrhines and platyrrhines (Poux et al. 2006; Loss-Oliveira et al. 2012).

Our estimates are evidently conditional on several parametric assumptions subject to further scrutiny. However, because the number of sites examined was statistically infinite, these estimates should be independent of additional genomic data for the species herein studied. Although our data set contained a number of statistically infinite nucleotide sites, credibility intervals of the time, and rate estimates were wide. This indicated that further precision of the timescale of NP evolution may only be achieved by augmenting the precision of the calibration based on the fossil record (Schrago and Voloch 2013). For example, the 95% HPD interval of the mean rate of molecular evolution varied from 0.9 to 1.9 × 10^−^^9^ s/s/y. This variation implies that, statistically, the age of the LCA of living NP ranged from 15.8 to 33 Ma. Although the probabilities of such marginal values are low, an alternative hypothesis of NP diversification proposing that the diversification of the three living families initiated at early as 30 Ma cannot be strictly discarded (Rosenberger 2011; Perez et al. 2013).

In coalescent-based analyses, a generation time of 10.3 years was assumed because there is no available estimate of the evolutionary rate per generation in NP. However, generation time varies as greatly as 6 years in *Callithrix* to 20 years in *Brachyteles* (Perez et al. 2013), and consequently, estimates of ancestral effective population size may also vary significantly. This variation would occur even if an analytical derivation were used. Moreover, because the genomic segments used in this study were sampled along the genome, it was expected that the rate of molecular evolution approached the neutral evolutionary rate. In great apes, the canonical neutral rate of 1.0 × 10^−^^9^ s/s/y is generally assumed. Although this estimate cannot be rejected statistically with our data because it lied within the credibility interval, there is evidence that evolutionary rates are inversely correlated with body size and endocranial volume in primates (Steiper and Seiffert 2012). It is therefore likely that the evolutionary rate of platyrrhines was actually higher that in great apes. It is worth mentioning that body size varies significantly between the three NP herein studied, and a reason why our estimated evolutionary rate is likely to be close to the average for NP.

Finally, the empirical distribution of gene trees deviated significantly from theoretical expectations under multispecies coalescent analyses. Theoretically, the frequency of the *Brachyteles*/*Callicebus* and *Callithrix*/*Callicebus* topologies should have been equal. However, such expectation would be only valid with free recombination between loci and also under a strict neutral model (Degnan and Rosenberg 2009). Because estimates from the full data set and from the clock-like data set were the same, the neutrality requirement has not been severely violated, although the assumption of free recombination might not be strictly valid when comparing the genomes of *B. arachnoides* (2n = 62) and *C. lugens* (2n = 16) because they represent the most extreme difference of linkage groups within NP. This difference, however, did not affect estimates of the ancestral effective population size because the frequency of the most likely gene tree (47%) used for calculating the ancestral *N*_e_ matched its theoretical expectation (48%).

In conclusion, analysis of a large sample of genomic sequences clarified the phylogenetics of New World anthropoid primates. The most likely tree topology along the phylome was sampled with a probability less than 50%. Thus, the majority rule consensus of the phylogenetic relationships of the three NP families results in an unresolved tree topology. This scenario is explained by the 2-fold expansion of the effective population size during the early diversification of NP. This rapid growth might be associated with the environmental changes in South America in the Early Miocene, specially in the Amazonian region, that suffered extensive geomorphological changes during this period.
